# High-efficiency backward four-wave mixing by quantum interference

**DOI:** 10.1038/s41598-017-16062-5

**Published:** 2017-11-17

**Authors:** Zi-Yu Liu, Jian-Ting Xiao, Jia-Kang Lin, Jun-Jie Wu, Jz-Yuan Juo, Chin-Yao Cheng, Yong-Fan Chen

**Affiliations:** Department of Physics, National Cheng Kung University, Tainan, 70101 Taiwan

## Abstract

Electromagnetically-induced-transparency-based four-wave mixing (FWM) in a resonant four-level double-Λ system has a maximum conversion efficiency (CE) of 25% due to spontaneous emission. Herein, we demonstrate that spontaneous emission can be considerably suppressed by arranging the applied laser beams in a backward configuration. With the backward double-Λ FWM scheme, we observe a CE of 63% in cold rubidium atoms with an optical depth (OD) of 48. To the best of our knowledge, this is the first observation of a CE exceeding the conversion limit in resonant FWM processes. Furthermore, we present a theoretical model that includes the phase-mismatch effect in the backward double-Λ FWM system. According to the theoretical model, the present scheme can achieve 96% CE using a medium with a large OD of 200 under ideal conditions. Such an efficient frequency conversion scheme has potential applications in optical quantum information technology.

## Introduction

Photons are suitable carriers for quantum information because of their fast propagation speed and weak interaction with the environment. Controlling the quantum states of individual photons with high fidelity is an important field of research in quantum information science. Another key topic is how to convert the frequencies of individual photons with little to no loss for transporting information among various types of quantum devices^1^. For instance, the low-loss wavelength for long-distance quantum communication in optical fibers is approximately 1550 nm, but it is more efficient to manipulate and detect the quantum state of light in the wavelength range of 600–800 nm for optical quantum information applications^2^. Such a photon frequency conversion technique is termed quantum frequency conversion (QFC)^3^. To date, QFC in the single-photon regime has been studied and discussed in various nonlinear optical systems, such as nonlinear crystals^4,5^, optical fibers^6,7^, and nanophotonic chips^8^. These nonlinear optical systems usually do not rely on a resonance transition. Thus, wave-mixing photons with various combinations of their frequencies obeying phase-match condition can produce emissions, causing the frequency of the converting photons to be spread over a large range and possess a large bandwidth. However, large photon bandwidth decreases the interaction strength between photon and matter, which increases the difficulty of implementing an efficient photon-photon gate and limits the practical applications of photonic quantum technologies^9^.

To overcome the problem of large photon bandwidth, a promising approach for implementing QFC with narrowband single photons is electromagnetically induced transparency (EIT)^10,11^. Single-photon-level nonlinear optics based on EIT has been widely studied^12–22^ because EIT can greatly suppress linear absorption and largely enhance nonlinear interactions. Recently, single-photon transistor has been realized in both cavity EIT^23^ and Rydberg EIT systems^24–26^. An all-optical *π*-order phase modulator has also been demonstrated at the photon level^27–30^. These EIT-based schemes have shown their potential in quantum information science and technology. Here, we focus on studying high-efficiency four-wave mixing (FWM) based on EIT with narrowband coherent light (approximately 1 MHz).

In 2004, resonant FWM based on double-Λ EIT scheme was first observed in cold atoms^31^. However, this resonant FWM scheme in a forward configuration has a maximum conversion efficiency (CE) of 25% due to the absorptive loss by spontaneous emission^32^. A simple method of suppressing spontaneous emission and thereby increasing the FWM efficiency in this forward double-Λ scheme is to shift the frequency of the applied laser field away from the resonant transition. Even though the interaction strength between light and matter is decreased in the non-resonant case, two research teams have demonstrated that the FWM efficiency can overcome the conversion limit (25%) of the resonant case^33,34^. Herein, we demonstrate that spontaneous emission can be effectively suppressed under a resonant condition only by arranging the applied laser beams in a backward configuration, which is the so-called backward FWM that was theoretically studied by Kang *et al*.^35^. With the backward double-Λ scheme, we observe an FWM efficiency of 63% in cold rubidium atoms with an optical depth (OD) of 48. To the best of our knowledge, this is the first observation of a CE exceeding 25% in the resonant double-Λ EIT scheme. According to the theoretical model, this resonant backward scheme can achieve 96% CE by using a medium with a large OD of 200, which reaches the same CE as the previous non-resonant scheme at half the OD^34^. Moreover, to compare the experimental data with the theoretical predictions, the phase-mismatch effect is included in the theoretical model, which was not considered in an earlier study of backward FWM^35^. We also note that FWM based on a double-Λ configuration has been extensively studied in hot atoms^36–44^ and atomic-like crystals^45^.

## Results and Discussion

### Experimental Details

**Figure 1:**
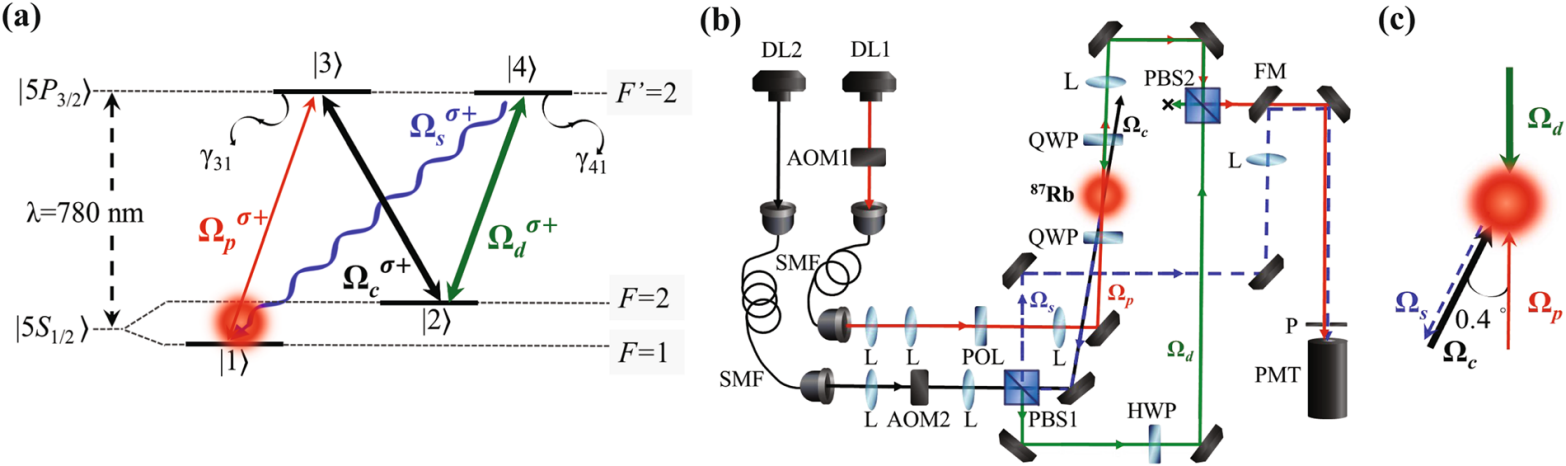
Energy level diagram and experimental arrangements. (**a**) Energy level of D_2_-line transition of ^87^Rb for EIT-based backward FWM. All fields are on resonance. (**b**) Schematic of the experimental setup. DL, diode laser; AOM, acousto-optic modulator; SMF, single-mode fiber; POL, polarizer; PBS, polarization beam splitter; HWP, half-wave plate; QWP, quarter-wave plate; L, lens; FM, flipping mirror; P, pinhole; PMT, photo-multiplier tube. (**c**) Relative-propagating direction arrangement of the optical beams. A small angle of approximately 0.4° is set between the probe and coupling beams. The driving beam is exactly counter propagating and coincides with the probe beam. The generated signal field is in the opposite direction of the coupling field, according to phase-match condition.

In the present study, we conduct the backward FWM experiment in a laser-cooled ^87^Rb atomic system. The relative energy levels and optical fields are shown in Fig. 1(a). All the cold atoms are initially prepared in the ground state $\mathrm{|1}\rangle$ by optical pumping. A weak probe field (Ω_*p*_ denotes its Rabi frequency) is on a resonance of $\mathrm{|1}\rangle \leftrightarrow \mathrm{|3}\rangle$ transition and forms a standard Λ-type EIT system with a strong coupling field (Ω_*c*_), which drives $\mathrm{|2}\rangle \leftrightarrow \mathrm{|3}\rangle$ transition. A driving field (Ω_*d*_) drives $\mathrm{|2}\rangle \leftrightarrow \mathrm{|4}\rangle$ transition and creates an FWM channel to convert the probe field into the signal field (Ω_*s*_). Both the $\mathrm{|3}\rangle$ and $\mathrm{|4}\rangle$ states are degenerated excited states and all the optical fields are right-circularly polarized (σ+).

The experimental setup is shown in Fig. 1(b). The probe field is produced from the diode laser (DL1), and the coupling and driving fields are generated by another diode laser (DL2). DL2 is directly injection locked by an external cavity diode laser, whereas DL1 is injection locked by an intermediate laser that is injection locked using the same external cavity diode laser through a 6.8-GHz electric-optical modulator output. Both the probe and coupling beams are sent to single-mode fibers (SMFs) to obtain spatial mode matching. After passing through the SMFs, the driving beam is portioned from the coupling beam by a polarization beam splitter. A half-wave plate is used to control the intensity of the driving beam. The *e* ^−2^ diameter of the probe beam waist is approximately 0.2 mm, whereas that of both the coupling and driving beams is approximately 2 mm.

The propagation direction and the relative position of the applied optical beams are depicted in Fig. 1(c). The coupling and probe beams are separated by a small angle of approximately 0.4° to reduce the light leakage from the coupling field. To conduct the backward FWM experiment, the driving field propagates from the opposite side of the cold atomic medium and is made to coincide with the probe field. Due to the phase-match condition, the propagation direction of the FWM signal field is the opposite of that of the coupling field [Fig. 1(c)]. The probe and signal pulses are detected by a photomultiplier tube module, and then recorded by an oscilloscope. The overall detection efficiencies for the probe and signal fields are approximately 25% and 10%, respectively, in the backward FWM experiment.

In the timing sequence, we denote the time of firing the probe pulse as *t* = 0 ms. The magnetic field of the magneto-optical trap (MOT) is first switched off at *t* = −1.5 ms. Next, we turn off the repumping laser of the MOT at *t* = −210 *μ*s and immediately turn on the coupling and the driving fields. The trapping laser of the MOT is switched off at *t* = −10 *μ*s before sending the probe pulse. This timing sequence is used to ensure that the entire atomic populations are prepared at the ground state $\mathrm{|1}\rangle$. The timings of all optical fields are controlled using individual acousto-optic modulators, and the repetition rate for each measurement is 100 Hz throughout the experiment. Note that to satisfy the condition that the spectral width of the probe pulse is much smaller than the width of the EIT transparency window, we set the duration of the probe square pulse to 40 *μ*s.

### Theoretical Model

We consider a four-level atomic system with two ground states ($\mathrm{|1}\rangle$ and $\mathrm{|2}\rangle$) and two excited states ($\mathrm{|3}\rangle$ and $\mathrm{|4}\rangle$), as shown in Fig. 1(a). The behavior of probe and signal pulses in the atomic medium under the backward FWM process are described by the Maxwell-Schrödinger equations (MSEs) as follows:

1 $$\frac{\partial {{\rm{\Omega }}}_{p}}{\partial z}+\frac{1}{c}\frac{\partial {{\rm{\Omega }}}_{p}}{\partial t}=i\frac{{\alpha }_{p}{\gamma }_{31}}{2L}{\rho }_{31},$$

2 $$-\frac{\partial {{\rm{\Omega }}}_{s}}{\partial z}+\frac{1}{c}\frac{\partial {{\rm{\Omega }}}_{s}}{\partial t}=i\frac{{\alpha }_{s}{\gamma }_{41}}{2L}{\rho }_{41},$$

where ${\rho }_{ij}$ represents the slowly varying amplitude of the coherence between $|i\rangle$ and $|j\rangle$, and ${\gamma }_{31}$ (${\gamma }_{41}$) represents the total coherence decay rates from the excited states $\mathrm{|3}\rangle$ ($\mathrm{|4}\rangle$). ${\alpha }_{p}=n{\sigma }_{13}L$ (${\alpha }_{s}=n{\sigma }_{14}L$) represents the OD of the probe (signal) transition, where *L* is the length of the medium. Note that ${\alpha }_{p}={\alpha }_{s}$ in this experiment because ${\sigma }_{13}={\sigma }_{14}$ when the degenerate Zeeman sublevels are considered. The minus sign of the space-derivative term in equation (2) indicates that the propagation direction of the signal pulse is the opposite of that of the probe pulse in the backward FWM case.

To solve equations (1) and (2), the equations of motion of ${\rho }_{13}$ and ${\rho }_{14}$ must be considered. When both the probe and signal fields are much weaker than the coupling and driving fields (i.e., ${\rho }_{11}=1$), the optical Bloch equations (OBEs) of the density matrix elements with slowly varying amplitude are given by

3 $$\frac{d}{dt}{\rho }_{41}=\frac{i}{2}{{\rm{\Omega }}}_{s}+\frac{i}{2}{{\rm{\Omega }}}_{d}{\rho }_{21}-\frac{{\gamma }_{41}}{2}{\rho }_{41},$$

4 $$\frac{d}{dt}{\rho }_{31}=\frac{i}{2}{{\rm{\Omega }}}_{p}+\frac{i}{2}{{\rm{\Omega }}}_{c}{\rho }_{21}-\frac{{\gamma }_{31}}{2}{\rho }_{31},$$

5 $$\frac{d}{dt}{\rho }_{21}=\frac{i}{2}{{\rm{\Omega }}}_{c}^{\ast }{\rho }_{31}+\frac{i}{2}{{\rm{\Omega }}}_{d}^{\ast }{\rho }_{41}-\frac{{\gamma }_{21}}{2}{\rho }_{21},$$

where ${\gamma }_{21}$ is the dephasing rate between ground states $\mathrm{|1}\rangle$ and $\mathrm{|2}\rangle$. Each parameter in the theoretical model is determined individually from additional experiments as follows: Ω_*c*_ is measured based on the separation of the two absorption peaks in the EIT spectrum^10,11^; OD is determined based on the delay time of the slow light pulse^46^; and ${\gamma }_{31}$ and ${\gamma }_{41}$ are mainly attributed to the spontaneous decay rate and laser linewidth (approximately 1 MHz) in the experiment, which are both roughly 1.25Γ according to the spectral width of the one-photon absorption. Γ = 2π × 6 MHz is the spontaneous decay rate of the excited states. Finally, in the backward FWM experiment, we determine the values of both Ω_*d*_ and ${\gamma }_{21}$ through numerical simulations of equations (1)–(5).

The analytic solutions of ${\rho }_{31}$ and ${\rho }_{41}$ can be obtained by solving equations (3)–(5). For simplicity, we assume that ${\gamma }_{21}=0$, ${\gamma }_{31}={\gamma }_{41}$, and ${\alpha }_{p}={\alpha }_{s}=\alpha$. With the time-derivative terms being zero, the steady-state solutions for ${\rho }_{31}$ and ${\rho }_{41}$ are as follows:

6 $${\rho }_{31}=\frac{|{{\rm{\Omega }}}_{d}{|}^{2}{{\rm{\Omega }}}_{p}-{{\rm{\Omega }}}_{s}{{{\rm{\Omega }}}_{d}}^{\ast }{{\rm{\Omega }}}_{c}}{D},$$

7 $${\rho }_{41}=\frac{|{{\rm{\Omega }}}_{c}{|}^{2}{{\rm{\Omega }}}_{s}-{{\rm{\Omega }}}_{p}{{{\rm{\Omega }}}_{c}}^{\ast }{{\rm{\Omega }}}_{d}}{D},$$

where $D=-i{\rm{\Gamma }}(|{{\rm{\Omega }}}_{c}{|}^{2}+|{{\rm{\Omega }}}_{d}{|}^{2})$. By setting boundary conditions of ${{\rm{\Omega }}}_{p}(z=\mathrm{0)}={{\rm{\Omega }}}_{p0}$ and ${{\rm{\Omega }}}_{s}(z=L)=0$ and substituting equations (6) and (7) into the MSEs [equations (1) and (2)] with the time-derivative terms being zero, we obtain the steady-state solutions for the probe and signal fields as follows:

8 $${{\rm{\Omega }}}_{p}(z=L)=\frac{(|{{\rm{\Omega }}}_{c}{|}^{2}-|{{\rm{\Omega }}}_{d}{|}^{2}){e}^{\xi }}{|{{\rm{\Omega }}}_{c}{|}^{2}{e}^{\xi }-|{{\rm{\Omega }}}_{d}{|}^{2}}{{\rm{\Omega }}}_{p0},$$

9 $${{\rm{\Omega }}}_{s}(z=\mathrm{0)}=\frac{{{{\rm{\Omega }}}_{c}}^{\ast }{{\rm{\Omega }}}_{d}\mathrm{(1}-{e}^{\xi })}{|{{\rm{\Omega }}}_{c}{|}^{2}{e}^{\xi }-|{{\rm{\Omega }}}_{d}{|}^{2}}{{\rm{\Omega }}}_{p0},$$

where $\xi =(\frac{\alpha }{2})\frac{|{{\rm{\Omega }}}_{c}{|}^{2}-|{{\rm{\Omega }}}_{d}{|}^{2}}{|{{\rm{\Omega }}}_{c}{|}^{2}+|{{\rm{\Omega }}}_{d}{|}^{2}}$ and ${{\rm{\Omega }}}_{p0}$ represents the incident probe field. Equations (8) and (9) are valid under the condition of $|{{\rm{\Omega }}}_{c}|\ne |{{\rm{\Omega }}}_{d}|$. When $|{{\rm{\Omega }}}_{c}|=|{{\rm{\Omega }}}_{d}|$, the solutions for the probe and signal fields have another expression as follows:

10 $${{\rm{\Omega }}}_{p}(z=L)=(\frac{4}{4+\alpha }){{\rm{\Omega }}}_{p0},$$

11 $${{\rm{\Omega }}}_{s}(z=\mathrm{0)}=(\frac{\alpha }{4+\alpha }){{\rm{\Omega }}}_{p0}\mathrm{.}$$

Thus, the steady-state CE of the backward FWM process under the condition of $|{{\rm{\Omega }}}_{c}|=|{{\rm{\Omega }}}_{d}|$ is expressed simply as follows:

12 $$\eta ={|\frac{{{\rm{\Omega }}}_{s}(z=\mathrm{0)}}{{{\rm{\Omega }}}_{p}(z=\mathrm{0)}}|}^{2}={(\frac{\alpha }{4+\alpha })}^{2}\mathrm{.}$$

According to equation (12), a 96% CE can be achieved in the resonant backward FWM through the use of a dense medium with a large OD of 200, overcoming the upper limit of 25% in the resonant forward case^35^. The reason for this increase in the CE can be inferred from equations (6) and (7). For the backward case, the intensity balance condition [i.e., ${{\rm{\Omega }}}_{p}(z){{\rm{\Omega }}}_{d}={{\rm{\Omega }}}_{s}(z){{\rm{\Omega }}}_{c}$] is satisfied the whole dense medium compared with the forward case, resulting in ${\rho }_{31}$ = ${\rho }_{41}$ = 0. Hence, the backward case automatically suppresses spontaneous emission in the whole dense medium and greatly improves the CE in resonant FWM processes.

### Phase-Mismatch Effect

We have discussed a theoretical model for the EIT-based backward FWM under ideal conditions. However, experiments usually involve some unwanted effects. In our experiment, the phase-mismatch effect cannot be neglected and should be included in the theoretical model. In our backward double-Λ scheme, the phase-mismatch term is expressed as follows:

13 $${\rm{\Delta }}\vec{k}=\mathop{{k}_{p}}\limits^{\longrightarrow}-\mathop{{k}_{c}}\limits^{\longrightarrow}+\mathop{{k}_{d}}\limits^{\longrightarrow}-\mathop{{k}_{s}}\limits^{\longrightarrow},$$

where the plus (minus) signs are determined by the fields that are absorbed (emitted) by the FWM medium. Owing to the backward configuration, $\mathop{{k}_{s}}\limits^{\longrightarrow}$ and $\mathop{{k}_{d}}\limits^{\longrightarrow}$ are in the opposite direction of $\mathop{{k}_{p}}\limits^{\longrightarrow}$ and $\mathop{{k}_{c}}\limits^{\longrightarrow}$, as shown in Fig. 1(c). If the phase-mismatch term, ${\rm{\Delta }}k$, is zero, the generated signal field in the backward FWM can be obtained from equation (11). However, if ${\rm{\Delta }}k\ne 0$, the phase-mismatch effect should be considered in the theoretical model. To include the phase-mismatch effect in the backward FWM, we can change the variable of ${{\rm{\Omega }}}_{s}={{\rm{\Omega }}^{\prime} }_{s}{e}^{i{\rm{\Delta }}kz}$, and the resulting MSEs from equations (1) and (2) and OBEs from equations (3)–(5) are modified as follows:

14 $$\frac{\partial {{\rm{\Omega }}}_{p}}{\partial z}+\frac{1}{c}\frac{\partial {{\rm{\Omega }}}_{p}}{\partial t}=i\frac{{\alpha }_{p}{\gamma }_{31}}{2L}{\rho }_{31},$$

15 $$-\frac{\partial {{\rm{\Omega }}^{\prime} }_{s}}{\partial z}+\frac{1}{c}\frac{\partial {{\rm{\Omega }}^{\prime} }_{s}}{\partial t}-i{\rm{\Delta }}k{{\rm{\Omega }}^{\prime} }_{s}=i\frac{{\alpha }_{s}{\gamma }_{41}}{2L}{\rho }_{41},$$

16 $$\frac{d}{dt}{\rho }_{41}=\frac{i}{2}{{\rm{\Omega }}^{\prime} }_{s}+\frac{i}{2}{{\rm{\Omega }}}_{d}{\rho }_{21}-\frac{{\gamma }_{41}}{2}{\rho }_{41},$$

17 $$\frac{d}{dt}{\rho }_{31}=\frac{i}{2}{{\rm{\Omega }}}_{p}+\frac{i}{2}{{\rm{\Omega }}}_{c}{\rho }_{21}-\frac{{\gamma }_{31}}{2}{\rho }_{31},$$

18 $$\frac{d}{dt}{\rho }_{21}=\frac{i}{2}{{\rm{\Omega }}}_{c}^{\ast }{\rho }_{31}+\frac{i}{2}{{\rm{\Omega }}}_{d}^{\ast }{\rho }_{41}-\frac{{\gamma }_{21}}{2}{\rho }_{21}\mathrm{.}$$

For simplicity, as we did before, we consider the conditions of ${\gamma }_{21}=0$, ${\gamma }_{31}={\gamma }_{41}$, ${\alpha }_{p}={\alpha }_{s}=\alpha$, and ${{\rm{\Omega }}}_{c}={{\rm{\Omega }}}_{d}$. After we solve the MSEs and OBEs under the phase-mismatch condition, the steady-state solutions of the probe and signal fields on resonance are given by

19 $${{\rm{\Omega }}}_{p}(z=L)=\frac{2\beta }{2\beta \,\cos (\frac{\beta }{2})+(\alpha -2i{\rm{\Delta }}kL)\sin (\frac{\beta }{2})}{{\rm{\Omega }}}_{p0}{e}^{-\frac{1}{2}i{\rm{\Delta }}kL},$$

20 $${{\rm{\Omega }}^{\prime} }_{s}(z=\mathrm{0)}=\frac{\alpha }{2\beta \,\cot (\frac{\beta }{2})+(\alpha -2i{\rm{\Delta }}kL)}{{\rm{\Omega }}}_{p0},$$

where $\beta =\sqrt{{({\rm{\Delta }}kL)}^{2}+i\alpha {\rm{\Delta }}kL}$. If ${\rm{\Delta }}k$ is small and approaches zero, phase mismatching reverts to phase matching, and equations (19) and (20) are altered to equations (10) and (11). Notably, equations (19) and (20) also predict that both the probe and signal fields acquire a phase shift from the phase-mismatch effect (${\rm{\Delta }}k\ne 0$) in the resonant FWM scheme.

### Experimental Observations

**Figure 2:**
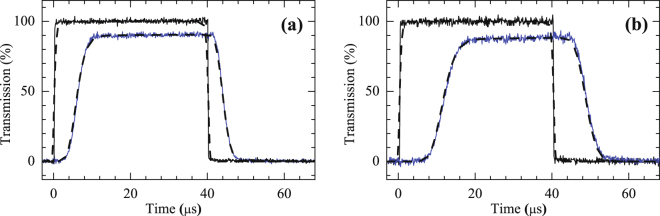
Observed slow-light effect based on EIT. The black (higher) and blue (lower) lines show the input probe pulse and the probe pulse after propagating through the medium, respectively. The solid lines represent the experimental data and the dashed lines represent the theoretical curves with the parameters (**a**) $\alpha =24$, ${{\rm{\Omega }}}_{c}=0.40{\rm{\Gamma }}$, and ${\gamma }_{21}=5\times {10}^{-4}{\rm{\Gamma }}$; (**b**) $\alpha =48$, ${{\rm{\Omega }}}_{c}=0.40{\rm{\Gamma }}$, and ${\gamma }_{21}=5\times {10}^{-4}{\rm{\Gamma }}$.

We first measure the slow light pulses due to the EIT effect, as shown in Fig. 2. The OD in Fig. 2(a) measured in a typical MOT is approximately 24. To further increase the OD, we measure the slow light pulse in a dark spontaneous-force optical trap (SPOT). Details on the experimental setup of the dark SPOT can be found in our previous study^20^. The OD in the dark SPOT, as shown in Fig. 2(b), is approximately 48, which is twice that in the MOT. Both of these experimental data are in good agreement with the theoretical curves (dashed lines). Moreover, the dephasing rate ${\gamma }_{21}$ is the same in the MOT and the dark SPOT ($5\times {10}^{-4}{\rm{\Gamma }}$).

**Figure 3:**
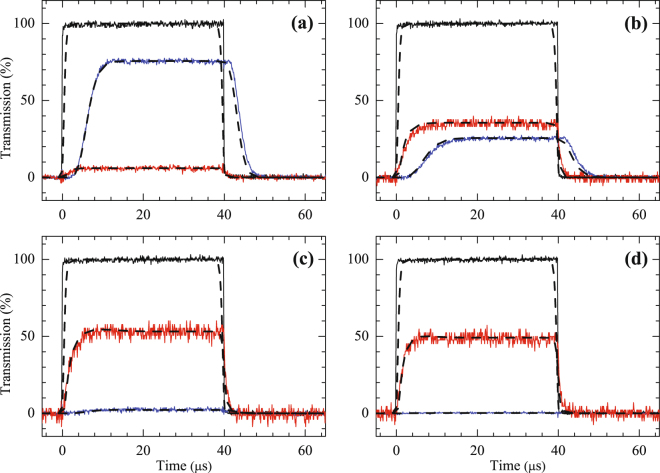
Observed probe and signal pulses propagating through the EIT-based backward FWM medium in the MOT system. The black lines represent the incident probe pulses. The red and blue lines represent the generated signal and the transmitted probe pulses, respectively. In the experiment, $\alpha =23$, ${{\rm{\Omega }}}_{c}=0.40{\rm{\Gamma }}$, and ${\rm{\Delta }}kL=0.364\pi$. The solid lines represent the experimental data and the dashed lines represent the theoretical curves with parameters (**a**) ${{\rm{\Omega }}}_{d}=0.10{\rm{\Gamma }}$ and ${\gamma }_{21}=8\times {10}^{-4}{\rm{\Gamma }}$; (**b**) ${{\rm{\Omega }}}_{d}=0.25{\rm{\Gamma }}$ and ${\gamma }_{21}=8\times {10}^{-4}{\rm{\Gamma }}$; (**c**) ${{\rm{\Omega }}}_{d}=0.37{\rm{\Gamma }}$ and ${\gamma }_{21}=1.6\times {10}^{-3}{\rm{\Gamma }}$; (**d**) ${{\rm{\Omega }}}_{d}=0.45{\rm{\Gamma }}$ and ${\gamma }_{21}=3.0\times {10}^{-3}{\rm{\Gamma }}$. According the theoretical curves, the steady-state CEs of the incident probe to the FWM signal are 6%, 36%, 53%, and 49% in (**a**–**d**), respectively.

After the OD is determined in the experiment, we turn on the driving field to perform the backward FWM experiment. Figure 3 shows the transmitted probe pulses (blue solid lines) and the FWM signal pluses (red solid lines) in the MOT system. We change the intensity of the driving field and measure the variation of CE in backward FWM processes, where the parameters of Ω_*d*_ are 0.10Γ, 0.25Γ, 0.37Γ, and 0.45Γ in Fig. 3(a–d), respectively. The phase-mismatch term ${\rm{\Delta }}k$ is calculated to be approximately 0.091 $\pi /mm\,$ according to the propagating directions of the probe, coupling, and driving fields mentioned in Fig. 1(c). Because the size of the cold atomic cloud in the MOT is measured to be approximately 4 mm, the value of ${\rm{\Delta }}kL$ is determined to be 0.364*π* in the experiment shown in Fig. 3. According to the theoretical prediction, the maximum CE in an ideal case (${\gamma }_{21}=0$) occurs when the Rabi frequency of the driving field is equal to that of the coupling field (i.e., ${{\rm{\Omega }}}_{c}={{\rm{\Omega }}}_{d}$). The experimental data in Fig. 3 are in good agreement with this theoretical prediction. A maximum CE of 53% is observed with ${{\rm{\Omega }}}_{d}=0.37{\rm{\Gamma }}$, as shown in Fig. 3(c), which is slightly smaller than ${{\rm{\Omega }}}_{c}=0.40{\rm{\Gamma }}$ due to the non-negligible ground-state dephasing rate in the experiment. To the best of our knowledge, this is the first experimental observation of a CE exceeding 25% in resonant double-$\Lambda$-based FWM processes. Furthermore, Fig. 3 reveals that the dephasing rate increases as the driving Rabi frequency increases, which implies that the EIT condition is slightly destroyed by the driving field coupling to the off-resonant excited state of $\mathrm{|5}{P}_{\mathrm{3/2}},F^{\prime} =3\rangle$. Although the frequency detuning of the driving field coupling to the off-resonant excited state is around 44 times the spontaneous decay rate, it causes a slight damage to the EIT condition through the far-detuning photon-switching effect^12^. This unwanted effect can be mitigated using the Rb^87^D_1_ transition with larger frequency splitting between the hyperfine energy levels compared with the D_2_ transition used in this experiment.

**Figure 4:**
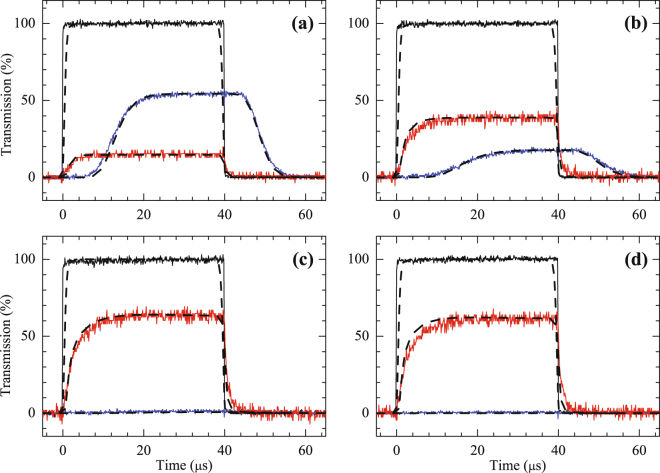
Observed probe and signal pulse propagating through the EIT-based backward FWM medium in the dark SPOT system. The black lines represent the incident probe pulses. The red and blue lines represent the generated signal and the transmitted probe pulses, respectively. In the experiment, $\alpha =48$, ${{\rm{\Omega }}}_{c}=0.40{\rm{\Gamma }}$, and ${\rm{\Delta }}kL=0.273\pi$. The solid lines represent the experiment data and the dashed lines represent the theoretical curves with parameters (**a**) ${{\rm{\Omega }}}_{d}=0.16{\rm{\Gamma }}$ and ${\gamma }_{21}=6\times {10}^{-4}{\rm{\Gamma }}$; (**b**) ${{\rm{\Omega }}}_{d}=0.26{\rm{\Gamma }}$ and ${\gamma }_{21}=1.1\times {11}^{-3}{\rm{\Gamma }}$; (**c**) ${{\rm{\Omega }}}_{d}=0.37{\rm{\Gamma }}$ and ${\gamma }_{21}=1.3\times {10}^{-3}{\rm{\Gamma }}$; (**d**) ${{\rm{\Omega }}}_{d}=0.43{\rm{\Gamma }}$ and ${\gamma }_{21}=2.3\times {10}^{-3}{\rm{\Gamma }}$. According the theoretical curves, the steady-state CEs of the incident probe to the FWM signal are 15%, 39%, 63%, and 62% in (**a**–**d**), respectively.

To pursue a higher CE, we next perform the backward FWM in the dark SPOT to produces cold atoms with a large OD of 48. The value of ${\rm{\Delta }}kL$ here is calculated to be 0.273*π* because the size of the cold atomic cloud in the dark SPOT is roughly 3 mm, which is smaller than that in the MOT. The transmitted probe and generated signal pulses propagating through the FWM medium are shown in Fig. 4. A maximum CE of 63% is observed when ${{\rm{\Omega }}}_{d}=0.37{\rm{\Gamma }}$, corresponding to a driving intensity of approximately 2 mW/cm^2^ in the experiment [Fig. 4(c)]. According to equation (12), this backward FWM scheme can achieve an 85% CE using a medium with an OD of 48 under ideal conditions (${\gamma }_{21}=0$ and ${\rm{\Delta }}k=0$). In this circumstance, equation (10) predicts that most of the probe pulse energy will be converted to the signal pulse by the FWM process, leaving only 1% energy in the probe pulse. Although approximately 14% of the energy of the probe pulse is lost through spontaneous emission, the CE in the backward case far exceeds the conversion limit of 25% in the forward case. If the phase-mismatch effect is not zero (${\rm{\Delta }}kL$ = 0.273*π*) but the ground-state dephasing rate is neglected (${\gamma }_{21}=0$) in our experiment, a maximum CE of 69% can be obtained from the backward FWM scheme, according to equation (20). In other words, approximately 16% of the energy of the generated signal pulse is lost due to the phase-mismatch effect in our experiment. However, in principle, the phase-mismatch effect in the double-Λ scheme can be compensated using small two-photon detuning^36^.

Figures 3 and 4 also indicate that the delay time of the generated signal pulse is much smaller than that of the transmitted probe pulse in the resonant FWM processes. This phenomenon can be explained as follows: The probe pulse propagating in the medium simultaneously interacts with the coupling and driving fields and then generates the signal pulse through the FWM process. Since the signal field is counter propagating to the probe, the part of the signal field generated at the leading edge of the medium can leave the medium much earlier than the probe field, as reflected in the faster rise of the signal pulse. By contrast, the part of the signal field generated at the trailing edge of the medium passes through the longer medium, leading to greater energy loss as reflected in the rapid decay of the signal pulse. Consequently, the group velocity of the signal pulse is faster than that of the probe pulse, even though Ω_*d*_ is much smaller than Ω_*c*_.

**Figure 5:**
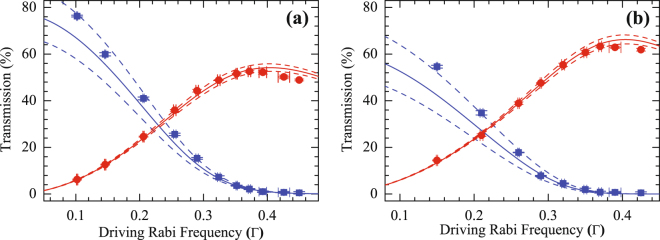
Transmission of the probe and the signal pulses propagating through the FWM medium as a function of driving Rabi frequency. The blue squares and red circles represent the probe and signal transmission, respectively. The experimental data shown in (**a**) and (**b**) are measured in the MOT and the dark SPOT system, respectively. The error bars represent $\pm 1$ standard deviation based on measurement statistics. The coupling Rabi frequency is fixed at 0.40Γ in the experiment. The solid lines show the theoretical curves with the parameters (**a**) $\alpha =23$, ${\gamma }_{21}=1.4\times {10}^{-3}{\rm{\Gamma }}$, and ${\rm{\Delta }}kL=0.364\pi$; (**b**) $\alpha =48$, ${\gamma }_{21}=1.3\times {10}^{-3}{\rm{\Gamma }}$, and ${\rm{\Delta }}kL=0.273\pi$. Considering all of the data in the measurement, ${\gamma }_{21}$ has a standard deviation of $8\times {10}^{-4}{\rm{\Gamma }}$ and $5\times {10}^{-4}{\rm{\Gamma }}$ in (**a**) and (**b**), respectively; hence, we also plot the theoretical curves (dashed lines) with the parameters (**a**) ${\gamma }_{21}=0.6\times {10}^{-4}{\rm{\Gamma }}$ (upper line) and ${\gamma }_{21}=2.2\times {10}^{-4}{\rm{\Gamma }}$ (lower line); (**b**) ${\gamma }_{21}=0.8\times {10}^{-4}{\rm{\Gamma }}$ (upper line) and ${\gamma }_{21}=1.8\times {10}^{-3}{\rm{\Gamma }}$ (lower line).

Figure 5 shows the transmission of the probe and signal pulses propagating through the FWM medium versus the driving Rabi frequency (Ω_*d*_). The OD in Fig. 5(a) and (b) are measured to be 23 $\pm$ 1 and 48 $\pm$ 2, respectively. The coupling Rabi frequency here is fixed at 0.40Γ. The blue squares (red circles) show the experimental data of the probe (signal) transmission. Both solid and dashed lines are the theoretical curves. The experimental data are in good agreement with the theoretical curves. For the FWM medium with an OD of 23, a maximum CE of 53% is observed when ${{\rm{\Omega }}}_{d}=0.37{\rm{\Gamma }}$, as shown in Fig. 5(a). Figure 5(b) reveals that the maximum CE is increased to 63% when the FWM medium with an OD of 48. According to equation (12), the maximum CE in the present scheme can further increase to 96% at an OD of 200, which is the same CE as that of the previous non-resonant FWM system but using only half the OD^34^. We also note that a different scheme has been proposed recently that uses the spatially varied intensity of two laser fields to overcome the conversion limit (25%) in resonant FWM processes; however this scheme has not yet been experimentally demonstrated^47^.

## Conclusion

In conclusion, we have obtained an EIT-based FWM efficiency of 63% by using the backward double-Λ scheme in cold rubidium atoms with an OD of 48. To the best of our knowledge, this is the first observation of a CE exceeding the conversion limit of 25% in resonant FWM processes. In addition, we present a theoretical model that includes the phase-mismatch effect in the backward double-Λ system. A comparison of the experimental data and the theoretical predictions shows good agreement. According to the theoretical model, the backward FWM scheme can greatly suppress spontaneous emission and reach nearly 100% CE by using a dense medium with a large OD under ideal conditions. Such a near-perfect frequency converter based on the EIT effect can convert frequency of narrowband photons, and hence has potential applications in optical quantum information technology.
